# Comparative analysis of the molecular and physiological consequences of constitutive SKN-1 activation

**DOI:** 10.1007/s11357-023-00937-9

**Published:** 2023-09-26

**Authors:** Carmen M. Ramos, Sean P. Curran

**Affiliations:** 1 Leonard Davis School of Gerontology, University of Southern California, Los Angeles, CA 90089 USA; 2 Dornsife College of Letters, Arts, and Sciences, Department of Molecular and Computational Biology, University of Southern California, Los Angeles, CA 90089 USA

**Keywords:** Cytoprotection, *skn-1*, Transcription, Aging, Proteostasis, *C. elegans*

## Abstract

Molecular homeostats play essential roles across all levels of biological organization to ensure a return to normal function after responding to abnormal internal and environmental events. SKN-1 is an evolutionarily conserved cytoprotective transcription factor that is integral for the maintenance of cellular homeostasis upon exposure to a variety of stress conditions. Despite the essentiality of turning on SKN-1/NRF2 in response to exogenous and endogenous stress, animals with chronic activation of SKN-1 display premature loss of health with age, and ultimately, diminished lifespan. Previous genetic models of constitutive SKN-1 activation include gain-of-function alleles of *skn-1* and loss-of-function alleles of *wdr-23* that impede the turnover of SKN-1 by the ubiquitin proteasome. Here, we define a novel gain-of-function mutation in the *xrep-4* locus that results in constitutive activation of SKN-1 in the absence of stress. Although each of these genetic mutations results in continuously unregulated transcriptional output from SKN-1, the physiological consequences of each model on development, stress resistance, reproduction, lipid homeostasis, and lifespan are distinct. Here, we provide a comprehensive assessment of the differential healthspan impacts across multiple models of constitutive SKN-1 activation. Although our results reveal the universal need to reign in the uncontrolled activity of cytoprotective transcription factors, we also define the unique signatures of each model of constitutive SKN-1 activation, which provides innovative solutions for the design of molecular “off-switches” of unregulated transcriptional homeostats.

**Supplementary Information:**

The online version contains supplementary material available at 10.1007/s11357-023-00937-9.

## Introduction

Homeostatic regulation is a vital component for the maintenance of cellular health. These controls play essential roles across all levels of biological organization to ensure a return to normal function after responding to abnormal internal and environmental events [1, 2]. Disruption of homeostatic regulators is a hallmark of aging and age-related pathologies [3, 4]. Cap’n’collar (Cnc) transcription factors represent a family of evolutionarily and functionally conserved proteins with central homeostatic functions [1, 4, 5]. Cnc proteins generally bind to antioxidant response elements (ARE) of genes encoding antioxidant and detoxification enzymes in the presence of oxidative stress [1, 5]. In mammalian systems, stressors such as reactive oxygen species (ROS), xenobiotics, and pathogens can activate the Cnc transcription factor NRF2 (nuclear factor-E2-related factor 2) stress response pathway [4, 6]. NRF2 is the most characterized of four members of the NFE2-related factors as it plays an integral role in coordinating phase II detoxification response [1, 4, 5, 7]. In *Caenorhabditis elegans,* the Cnc transcription factor Skinhead-1 (SKN-1) is the evolutionarily conserved cytoprotective transcription factor that is homologous to NRF, as both share similarities in their DNA-binding region and a conserved DIDLID motif element [1, 4, 8, 9]. Four distinct isoforms are encoded by the *C. elegans skn-1* gene, with three isoforms sharing the C-terminal DNA binding domain [1, 7]. SKN-1a and SKN-1c isoforms, corresponding most similarly to NRF1 and NRF2, respectively, accumulate and function in the intestine to mediate detoxification and stress responses [1, 7, 8, 10]. Isoform SKN-1b has been shown to be expressed in the ASI chemosensory neurons and is suggested to serve as a food-sensor to modulate metabolic homeostasis and longevity [1, 11]. SKN-1/NRF2 are integral for the maintenance of cellular homeostasis through phase II detoxification pathways upon exposure to a variety of stress conditions [1, 8, 9, 12, 13]. In addition to roles in detoxification responses, SKN-1 is essential for early development as embryos of null allele mutants are lethal [1, 14, 15]. The activation of this cytoprotective transcription factor is essential for the cells ability to maintain homeostasis in response to stress [9, 16–18].

Despite the essentiality of turning on SKN-1/NRF2 in response to exogenous and endogenous stress, animals with chronic activation of SKN-1 display premature loss of health with age, and ultimately, diminished lifespan [1, 8, 16, 19, 20]. This work has demonstrated that turning off cytoprotection is equally as vital as the ability to turn it on to avoid detrimental outcomes. Previous genetic models of constitutive SKN-1 activation include loss-of-function alleles of *wdr-23* and gain-of-function alleles of *skn-1* that impede the turnover of SKN-1 by the ubiquitin proteasome [6, 9, 21]. WDR-23 acts as the direct repressor of SKN-1 activity by targeting SKN-1 for degradation by the ubiquitin proteosome [21–25]. As such, in the absence of *wdr-23*, SKN-1 is stabilized and translocated into the nucleus where it can bind and induce the transcriptional activity of genes that will mitigate stress conditions [21–25]. *wdr-23lf* mutants display an increase in oxidative stress resistance, an upregulation in stress response, metabolic, and signaling genes, and lifespan extension [7, 24, 25]. Subsequently, there is a transcriptional redirection in gain-of-function (*gf*) mutations in *skn-1* that mediates altered survival responses [16]. Here, the *skn-1gf* allele (*lax188)* used does not alter the SKN-1b polypeptide but is predicted to modulate the activity of SKN-1a and SKN-1c isoforms [1, 7]. These constitutively active SKN-1 mutants exhibit age-dependent phenotypes, with an increase in stress resistance early in life, but later display an age-dependent somatic depletion of fat (Asdf) phenotype where somatic lipids are depleted and mobilized to the germline, ensuring reproductive fitness [16, 19]. Therefore, chronic activation of SKN-1 drives the dysregulation of cellular lipid homeostasis, diminished health, and altered lifespan [16, 19, 20].

Constitutive activation of NRF2 in mammalian cancer patients contributes to cancer cell proliferation and enhances chemoresistance through phase II detoxification pathways that induce activity of pro-survival genes [1, 4, 26]. The strong evolutionary conservation of these genes suggests these studies will inform fundamental questions in cell biology that are also clinically relevant, as unregulated NRF2 has recently been shown to be overactive in several chemo-resistant and radiation-resistant cancers [4, 5, 23, 26]. While each of these genetic mutations results in continuous unregulated transcriptional output from SKN-1, the physiological consequences of each model on health and lifespan metrics are distinct. Nevertheless, the full impact of constitutive SKN-1/NRF2 activation and its pleiotropic outcomes remains paradoxical and requires further exploration. Although many studies have uncovered that the activation of cytoprotective transcription factors can be detrimental if left unchecked, the complexity of homeostatic regulation to ensure survival in these conditions is not well understood.

Here, we define a novel gain-of-function mutation in the *xrep-4* locus that results in the constitutive activation of wild type SKN-1 in the absence of exogenous stress. The *xrep-4* gene encodes an F-box protein that interacts with and regulates the activity of WDR-23 [22, 25]. We provide a comprehensive assessment of the differential impact of constitutive SKN-1 activation across genetic models. Our data provides insight for the design of molecular “off-switches” to disrupt unregulated transcriptional output.

## Materials and methods

### *Caenorhabditis elegans* strains and maintenance

*Caenorhabditis elegans* were raised on 6 cm nematode growth media (NGM) plates supplemented with streptomycin and seeded with *E. coli* B strain OP50. All worm strains were grown using standard techniques at 20°C and unstarved for at least three generations before being used. Strains used in this study include: N2 Bristol (wild type), Hawaiian CB4856, CL2166, *gst-4p::GFP*, SPC168, *gst-4p::GFP;skn-1gf(lax188)*; SPC227, *skn-1gf(lax188)*; SPC131, *gst-4p::GFP;xrep-4gf* (*lax137)*; SPC597, *xrep-4gf(lax137)*.

### EMS mutagenesis screen and InDel mapping

Ethyl methanesulfonate (EMS) mutagenesis was performed as previously described [9]. Briefly, *gst-4p::GFP* was mutagenized with EMS for 4 h at 20°C and dropped on NGM plates seeded with OP50. F1 offspring from the mutagenized P0 with high GFP expression (indicating dominant activation of SKN-1) were selected. Isolated mutant was crossed into the polymorphic Hawaiian CB4856 strain, and the *xrep-4gf(lax137)* strain was isolated and genetically mapped to chromosome I as previously described [6, 9, 28–30] by using the InDel mapping primer set [31]. Whole-genome sequencing and injection rescue confirmed mutant sequence identity within the *F46F11.6* (*xrep-4)* locus. Mutants were outcrossed to wild type N2 to remove background mutations before being used for experiments.

### Whole-genome sequencing

Whole-genome sequencing (WGS) was performed as previously described [29]. Briefly, worms were egg prepped and eggs were allowed to hatch overnight. The next day ~4000 synchronized L1s were dropped on NGM plates seeded with 25X concentrated OP50. After 48 h, L4 animals were washed three times with M9, homogenized, and genomic DNA was extracted using Zymo Quick-DNA Miniprep Kit (Cat. #D3025). DNA samples were library prepped and sequenced by Novogene. Sequencing data were analyzed using Galaxy workflow: Bowtie2 (Galaxy Version 0.4), Filter (Galaxy Version 0.0.1), MPileup (Galaxy Version 2.0), VarScan (Galaxy Version 2.4.2) and R package DESeq2.

### RNAi treatment

RNAi treatment was performed as previously described [46]. Briefly, HT115/K-12 bacteria containing specific double-stranded RNA-expression plasmids were inoculated overnight in Luria broth (LB) containing selective antibiotic, seeded on NGM plates containing 5mM isopropyl-β-D-thiogalactoside and 50μg ml^-1^ carbenicillin. RNAi was induced at room temperature for 24 h. Synchronized L1 animals were placed on those plates to knockdown indicated genes.

### Hydrogen peroxide treatment

Hydrogen peroxide (H_2_O_2_) treatment was performed as previously described [16]. Briefly, synchronous worm populations at day 1 adulthood were washed 3x with M9 + 0.01% Triton. 500uL of 20mM H_2_O_2_ was then added to 600uL of worms + M9 + 0.01% Triton for a final concentration of 10mM. Worms were then incubated on a rotator at 20°C for 25 minutes before being rinsed 3x with M9 + 0.01% Triton and placed on a seeded plate. Chronic survival was measured 24 h later.

#### Nile Red staining

Nile Red fat staining was conducted as previously outlined in Stuhr et al. [48]. In brief, worms were egg prepped and allowed to hatch overnight for a synchronous L1 population. The next day, worms were dropped onto plates seeded with bacteria and raised to 48 h (L4 stage). Worms were washed off plates with PBS + 0.01% Triton, rocked for 3 min in 40% isopropyl alcohol before being pelleted and fixed with Nile Red in 40% isopropyl alcohol for 2 h. Worms were pelleted after 2 h and washed in PBS + 0.01% Triton for 30 min before being imaged at 10× magnification with ZEN Software and Zen Axio Imager with the DIC and GFP filter. Fluorescence is measured via corrected total cell fluorescence (CTCF) via ImageJ and Microsoft Excel. CTCF= (Worm Integrated Density – Background Integrated Density)/Worm area and normalized to the control.

### Oil Red O staining

Oil Red O fat staining was conducted as previously outlined in Stuhr et al. [48]. In brief, worms were egg prepped and allowed to hatch overnight for a synchronous L1 population. The next day, worms were dropped onto plates seeded with bacteria and raised to 120 h (day 3 adult stage) or 168 h (day 5 adult stage). Worms were washed off plates with PBS + 0.01% Triton, then rocked for 3 min in 40% isopropyl alcohol before being pelleted and treated with ORO in diH_2_O for 2 h. Worms were pelleted after 2 h and washed in PBS + 0.01% Triton for 30 min before being imaged at 20× magnification with LAS X software and Leica Thunder Imager Flexacam C3 color camera.

### Asdf quantification

ORO-stained worms were placed on glass slides and a coverslip was placed over the sample. Worms were scored as previously described [48]. Worms were scored and images were taken with LAS X software and Leica Thunder Imager Flexacam C3 color camera. Fat levels of worms were placed into three categories: non-Asdf, intermediate, and Asdf. Non-Asdf worms display no loss of fat and are stained dark red throughout most of the body (somatic and germ cells). Intermediate worms display significant fat loss from the somatic tissues, with portions of the intestine being clear, but ORO-stained fat deposits are still visible (somatic < germ cells)*.* Asdf worms had most, if not all, observable somatic fat deposits depleted (germ cells only) or significant fat loss from the somatic tissues with portions of the intestine being clear (somatic < germ).

### WormLab measurements

Worms were egg prepped and eggs were allowed to hatch overnight for a synchronous L1 population. The next day, worms were dropped onto plates seeded with OP50. Worms were then allowed to grow until each time point (48 h post-drop for L4s, 72 h post-drop for day 1 adults, and 120 h post-drop for day 3 adults). Once worms were the required stage of development, 30–50 worms were washed off a plate in 50 uL of M9 with a M9 + 0.01% Triton coated P1000 tip and dropped onto an unseeded NGM plate. The M9 was allowed to dissipate, and worms roamed on the unseeded plate for 1 hour before imaging crawling. Crawling was imaged with the WormLab microscope (MBF Bioscience), and analysis was performed with WormLab version 2022. Worm crawling on the plate was imaged for 1 minute for each condition at 7.5 ms. Worm crawling was analyzed with the software and only worms that moved for at least 90% of the time were included in the analysis.

### Lifespan analysis

Lifespan assays were performed as previously described [28, 29]. Briefly, worms were egg prepped and eggs were allowed to hatch overnight. The next day synchronized L1 larvae were dropped on NGM plates seeded with OP50. 48 h later, approximately 50 L4 hermaphrodites were moved individually onto new plates in replicates of three for each genotype. Worms were kept at 20°C and transferred every other day during the reproductive period. Worms were scored daily for survival by gentle prodding with a platinum wire. Worms that died of vulva burst, bagging, or crawling to the sides of plates were censored and discarded.

### RNA sequencing

RNAseq analysis was conducted as outlined in Stuhr and Curran [45]. Worms were egg prepped and eggs were allowed to hatch overnight for a synchronous L1 population. The next day, L1s were dropped onto seeded NGM plates and allowed to grow 48 h (L4 stage) before collection. Animals were washed 3 times with M9 buffer and frozen in TRI reagent at −80°C until use. Animals were homogenized and RNA extraction was performed via the Zymo Direct-zol RNA Miniprep kit (Cat. #R2052). Qubit™ RNA BR Assay Kit was used to determine RNA concentration. The RNA samples were sequenced and read counts were reported by Novogene. Read counts were then used for differential expression (DE) analysis using the R package DESeq2 created using R version 3.5.2. Statistically significant genes were chosen based on the adjust p-values that were calculated with the DESeq2 package. Genes were selected if their p value <0.01. Gene Ontology was analyzed using the most recent version of WormCat 2.0 [62].

## Results

### Identification of a novel *xrep-4* gain-of-function mutation

**Fig. 1:**
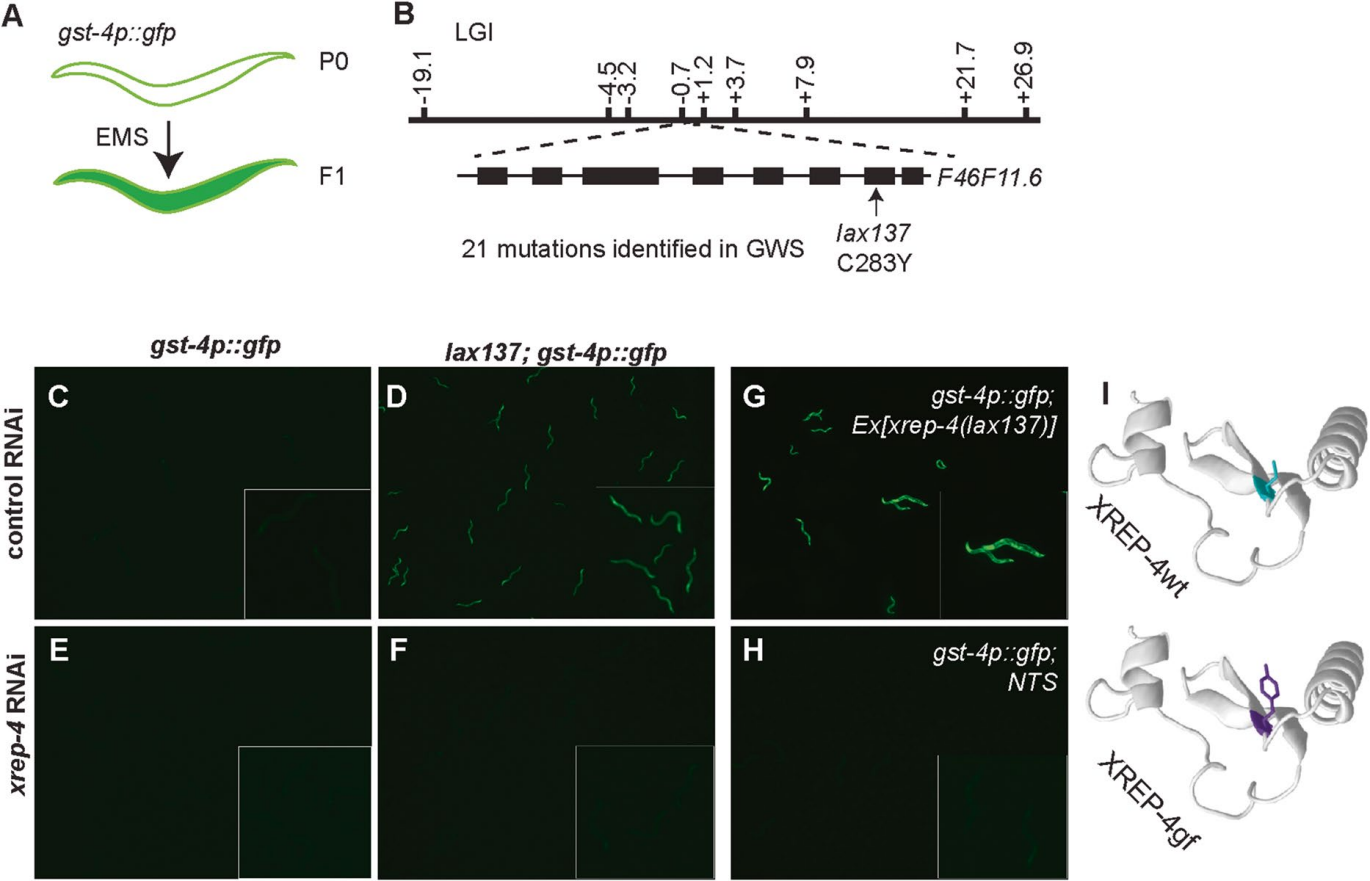
Identification of *xrep-4gf* mutation that induces *gst-4* expression. **A** Cartoon model of EMS screen for activators of the *gst-4p::GFP* reporter. **B** Whole-genome sequencing and InDel mapping identifies genetic linkage of *lax137* to the middle of chromosome I, as marked by dashed lines. Amino acid substitution in *xrep-4gf(lax137)* marked by the arrow.** C–F** RNAi of *xrep-4* suppresses the observed *gst-4p::GFP* activation in *lax137;gst-4p::GFP* animals. **F**. Ectopic expression of *xrep-4gf* phenocopies the increased expression of *gst-4p::GFP* (**G**) as compared to non-transgenic siblings (NTS) (**H**). **I** Phyre2 predicted structure of XREP-4 WT (cyan), with amino acid substitution in *xrep-4gf* (purple)

During phase II detoxification, SKN-1 activation mediates the transcription of glutathione S transferase (*gst*) genes which encode enzymes that catalyze the conjugation of reduced glutathione to electrophilic xenobiotics [1, 8, 9, 27]. To uncover regulators of the SKN-1/NRF2 homeostatic stress response network, a forward genetic screen with ethyl methanesulfonate (EMS) was performed on a *gst-4p::GFP* reporter strain, as a surrogate for SKN-1 activation (Fig. 1A) [9, 20]*.* From this screen, several classes of mutants were previously isolated including loss-of-function (*lf*) mutations in the Cul4 substrate receptor *wdr-23* [6, 21] and the mitochondrial proline catabolism pathway gene *alh-6* [20, 28–30], and gain-of-function (*gf*) mutations in the *skn-1* gene [9, 16].

Here, we isolated a second dominant mutation, *lax137*, that can activate the *gst-4p::GFP* reporter. We used the dominant induction of GFP expression to establish linkage of this mutant allele between −3.2 and +21.7 on LGI (Fig. 1B) [31]. We employed whole-genome sequencing (WGS) which revealed 25 non-synonymous mutations in the exons of protein-coding genes (Table S1) in this region on chromosome I. Subsequently, we performed an RNA interference (RNAi) screen of each of these genes in the *lax137;gst-4p::GFP* strain and looked for reversal of the GFP phenotype once the expression of the dominant mutant allele was inhibited. Only RNAi of *F46F11.6*, which encodes the F-box protein-encoding gene *xrep-4*, restored GFP expression to wild type (WT) levels (Fig. 1C–F and Figure S1A). To confirm causality, we generated transgenic animals harboring an extrachromosomal array of the *xrep-4(lax137)* gene, which phenocopied the high levels of GFP fluorescence from the *gst-4p::GFP* reporter and simultaneously established the gain-of-function nature of the *lax137* mutation (Fig. 1G, H). The *lax137* allele contains a cysteine (C) to tyrosine (Y) amino acid substitution in exon 7 of *xrep-4* at position 283 (C283Y) [32, 33] (Fig. 1I). The F-box domain in XREP-4 is predicted to lay at the N-terminal side of the protein and include amino acids 16-50 and as such, it is unlikely that the C283Y mutation alters protein-protein interactions mediated by this region. Taken together, these results reveal a dominant and gain-of-function mutation in the *F46F11.6*/*xrep-4* locus that we hereafter referred to as *xrep-4gf.*

### The transcriptional landscape of *xrep-4gf* mutants resembles animals with activated SKN-1

**Fig. 2:**
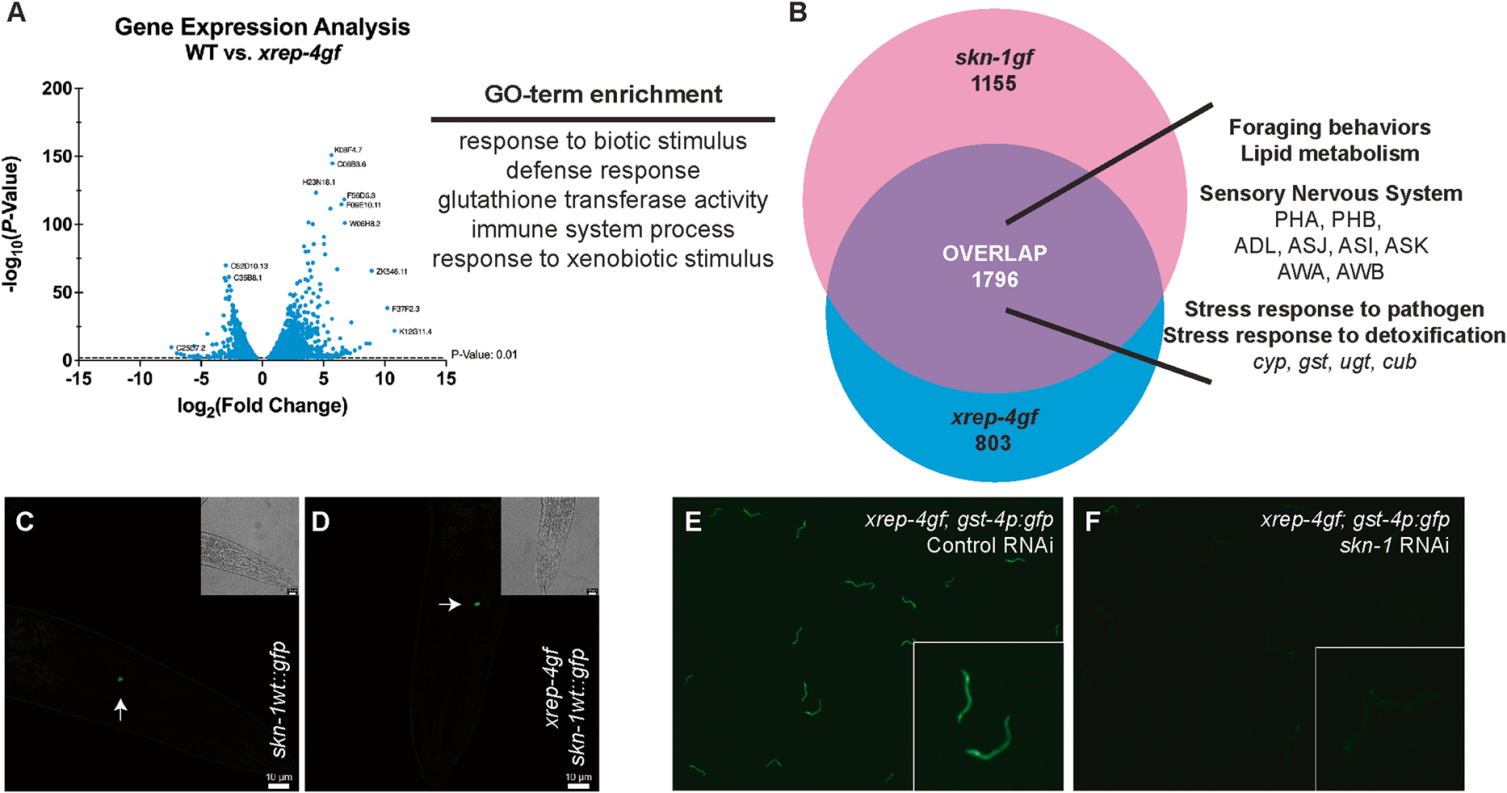
*xrep-4gf* mutants activate SKN-1 transcription. **A** Volcano plot of differentially expressed genes in *xrep-4gf* mutants compared to wild type (WT) animals, all genes have a *p*-value < 0.01. GO-term enrichment of *xrep-4gf* genes are associated with activated SKN-1. **B** 1796 genes are upregulated in both *xrep-4gf* and *skn-1gf* mutants that are associated with expression in foraging behaviors, lipid metabolism, and the sensory nervous systems. SKN-1wt-GFP expression is restricted to ASI neurons (arrows) in WT (**C**) and *xrep-4gf* mutants (**D**). As compared to control RNAi (**E**), RNAi of *skn-1* reduces *xrep-4gf(lax137)* activation of the *gst-4p::gfp* reporter (**F**)

To determine the transcriptional effects of the *xrep-4gf* mutation we performed RNAseq of L4-stage animals and compared to stage-matched wild type animals (Table S2). An analysis of classes of genes with significant increase in expression (>2-fold and p<0.01) revealed enrichment in genes with GO-terms associated with response to biotic stimulus, defense response, glutathione transferase activity, immune system process, and response to xenobiotic stimulus (Fig. 2A). Each of these GO-terms are associated with activation of SKN-1 [1, 9, 16, 19, 20, 24, 27, 34–40] and as such we performed an additional computational comparison with the transcriptional profile of *skn-1gf* mutants (Fig. 2B and Table S3). In addition to canonical SKN-1 regulated genes involved in cellular detoxification and pathogen responses (e.g., *gst*, *cyp*, *ugt*, *cub*), gene set enrichment analysis of the overlap between *xrep-4gf* and *skn-1gf* mutants revealed genes associated with expression in the sensory nervous system, specifically the PHA, PHB, ADL, ASJ, ASI, ASK, AWA, and AWB ciliated neurons, and also foraging behaviors and lipid metabolism. To test the impact of *xrep-4gf* mutation on SKN-1 protein stability, we examined the localization of a strain harboring a CRISPR-Cas9 engineered GFP tag at the C-terminus of the endogenous *skn-1* locus on wild type animals, referred to as SKN-1wt-GFP. This endogenously tagged SKN-1 revealed that, similar to other activation models, the expression of SKN-1wt-GFP is only visible in the ASI neurons (Fig. 2C, D). Lastly, RNAi targeting *skn-1* in the *xrep-4gf; gst-4p::GFP* strain reduced the increased GFP fluorescence to WT levels (Fig. 2E, F). Taken together these data demonstrate that *xrep-4gf* mutants results in the activation of SKN-1-dependent transcription.

### *xrep-4gf* animals are a phenotypically distinct model for constitutive SKN-1 activation

**Fig. 3:**
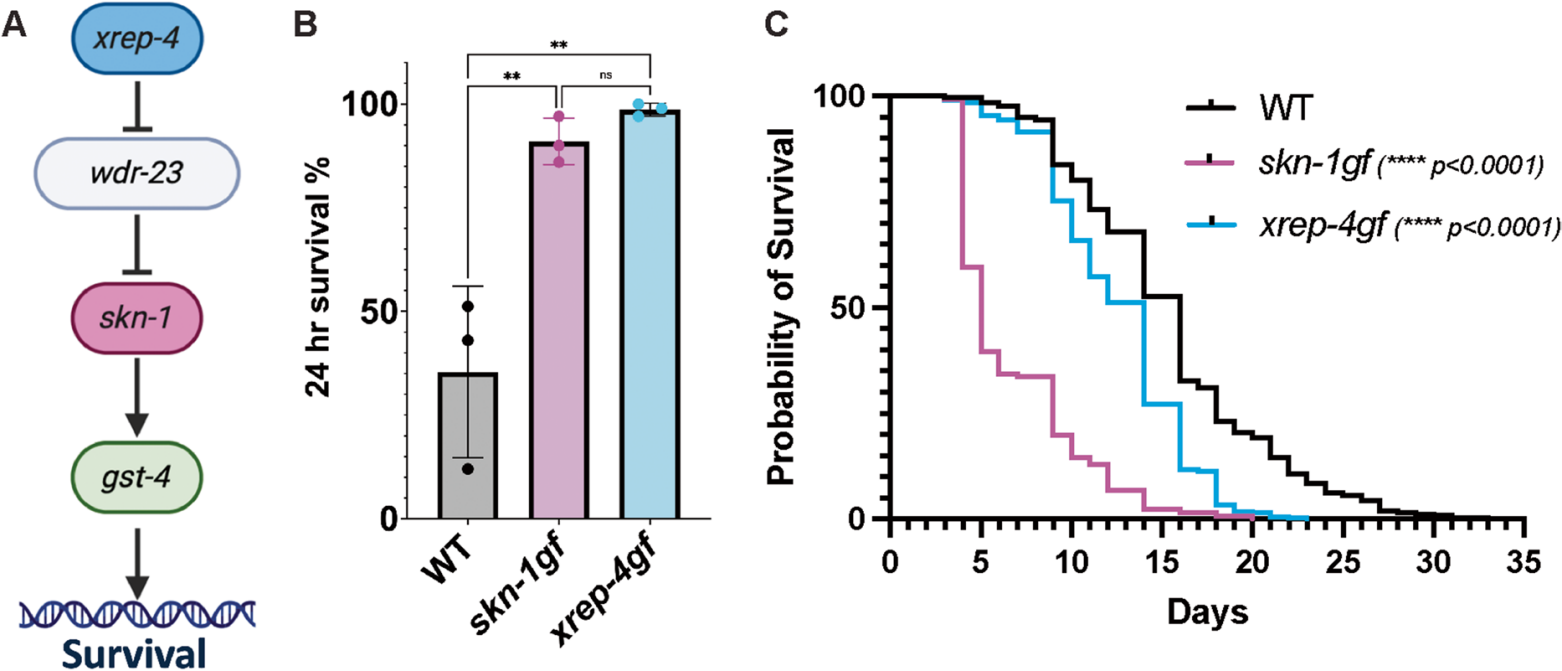
Models of constitutive SKN-1 activation. **A** Genetic pathway for *xrep-4* regulation of *skn-1* survival responses. **B** *skn-1gf* and *xrep-4gf* mutants at day 1 adulthood display enhanced resistance to acute hydrogen peroxide exposure. Statistical comparisons by one-way ANOVA test. **p < 0.005. *n=*150–200;* N*=3 per condition. **C** *skn-1gf* and *xrep-4gf* animals display shortened maximal lifespan (4th quartile) relative to WT, while *xrep-4gf* animals have a median lifespan more similar to WT controls

Previous work has described the role of XREP-4 in the negative regulation of WDR-23 [22, 23, 25], which in turn negatively regulates SKN-1 [7, 21, 24, 41–43], and as such, our *xrep-4gf* mutant is a powerful model to study SKN-1 activation (Fig. 3A). SKN-1 is integral for regulating resistance against oxidative stressors as animals missing functional *skn-1* are sensitive to exposure of oxidants and animals with constitutive activation of SKN-1 are resistant [7, 8, 16, 23, 43]. Similarly, *xrep-4gf* mutants display significant resistance to acute exposure to hydrogen peroxide (H_2_O_2_) at day 1 of adulthood as compared to WT, and that is indistinguishable from *skn-1gf* mutants and revealing that *xrep-4* function is important for survival in stress resistance (Fig. 3B).

Although activation of SKN-1 is important for responses to acute stress, chronic and unregulated activation of SKN-1 can reduce longevity [9, 16, 19]. To test the impact of constitutive activation of XREP-4, we assessed the lifespan of WT, *xrep-4gf*, and *skn-1gf* mutants under standard culturing conditions [30, 44–46] (Fig. 3C and Table S4). Similar to *skn-1gf* mutant animals, the maximal lifespan (last quartile) of the *xrep-4gf* mutants was reduced relative to WT animals. Intriguingly, *xrep-4gf* mutant animals have a more than double median lifespan, with similar survival statistics as WT animals at the first and second quartiles, which suggest similar rates of aging to midlife without the premature loss of health that is observed in *skn-1gf* mutants.

**Fig. 4:**
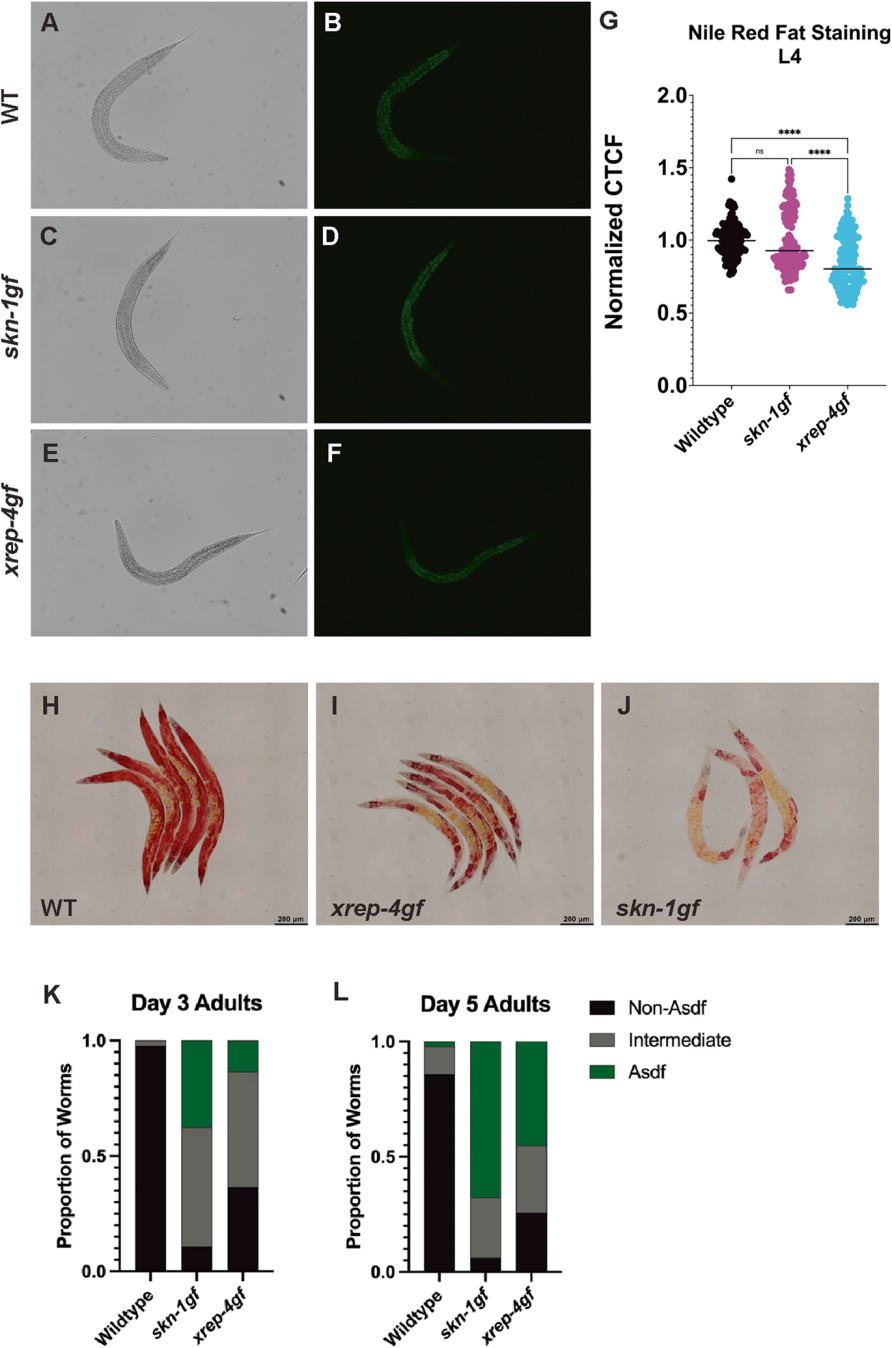
*xrep-4gf* mutants display age-dependent somatic depletion of fat (Asdf). **A**–**F** Representative images of Nile Red fixed-staining of age-matched L4-stage animals and total stored lipids quantified **G** Statistical comparisons by one-way ANOVA test. ****p < 0.0001. *n*=50;* N*=3 per condition. Intracellular lipid distribution in day 3 constitutive SKN-1 activation mutants via Oil Red O (ORO) staining (**H**–**J**) and quantification of the percentage of the population displaying non-Asdf, intermediate, and Asdf phenotypes at day 3 (**K**) and day 5 (**L**) of adulthood. *n*=50–100;* N*=3 per condition. CTCF: corrected total cell fluorescence

We next tested the physiological basis of the relatively normal early rate of aging in the *xrep-4gf* mutant animals that ultimately ends in a shortened maximal lifespan of the cohort. The loss of health in *skn-1gf* mutants is tied to organismal lipid homeostasis over the lifespan [16, 19]. With this in mind, we looked at the abundance and distribution of intracellular lipid stores in *xrep-4gf* mutants with Nile Red (NR) and Oil Red O (ORO), respectively (Fig. 4 and Table S5) [47, 48]. Relative to WT animals (Fig. 4A, B), *skn-1gf* mutants have similar overall lipid stores (Fig. 4C, D) but *xrep-4gf* mutant animals display a significant reduction in stored lipids (Fig. 4E, F). Animals with activated SKN-1 also display an age-dependent somatic depletion of fat (Asdf) phenotype that is connected to both stress resistance and survival [16, 19]. Unlike WT animals that maintain somatic lipid pools (Fig. 4H), *xrep-4gf* (Fig. 4I) and *skn-1gf* mutants (Fig. 4J) display Asdf. Notably, *xrep-4gf* mutants are slower to deplete somatic lipids as compared to *skn-1gf* mutants (Fig. 4K, L), which correlates with the improved health of *xrep-4gf* mutants up until mid-life, but the eventual indistinguishable shortening of maximal lifespan.

### Differential accelerated decline in health with age in different models of SKN-1 activation

**Fig. 5:**
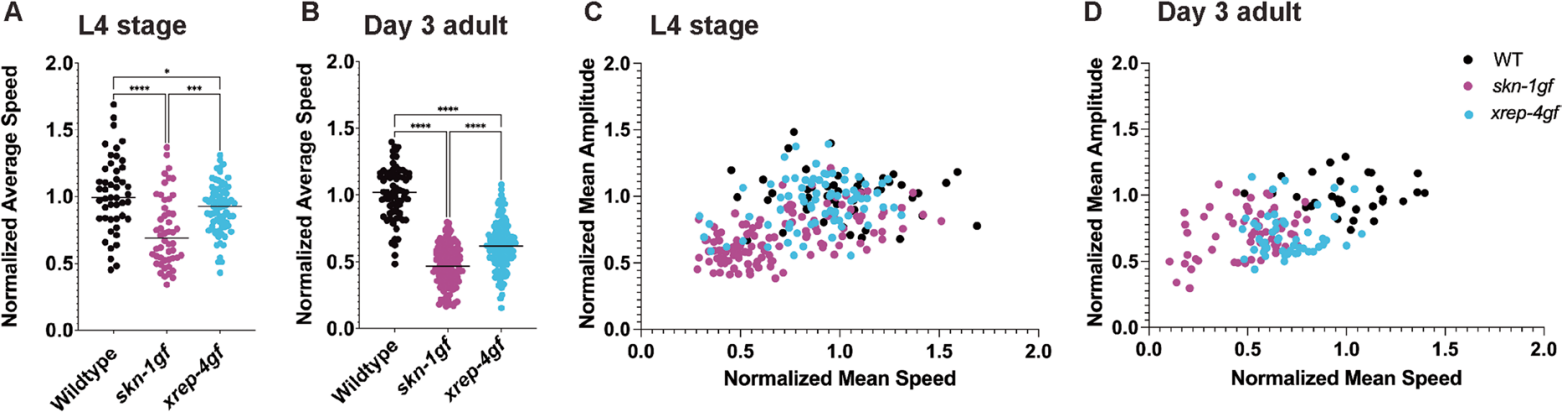
Constitutive activation of SKN-1 drives premature loss of muscle function. **A**–**D** Movement is progressively impaired in *skn-1gf* and *xrep-4gf* mutants at L4 and day 3 of adulthood*.* Statistical comparisons by one-way ANOVA test. *p<0.05, *** p<0.0005, **** p<0.0001. *n=*30–50;* N*=3 per condition

We next tested whether the *xrep-4gf* model of SKN-1 activation had additional effects on animal health. Beyond lipid homeostasis defects observed in *skn-1gf* mutants, these animals also display an accelerated age-related decline in motility (Fig. 5A, B and Table S6). *xrep-4gf* mutants also present with premature decline in crawling speed, but less severe than *skn-1gf* mutants at L4 stage (Fig. 5A) and day 3 of adulthood (Fig. 5B). Previous studies of *wdr-23* mutants, which activate SKN-1, show significantly reduced roaming when compared to wild type animals [7] and as such we examined roaming vs. dwelling behavior, which compares crawling speed to amplitude of body bends, of *xrep-4gf* and *skn-1gf* animals relative to age-matched WT controls (Fig. 5C, D). Like the previous age-related changes measured, *xrep-4gf* mutants display an intermediate phenotype relative to *skn-1gf* and WT animals.

Taken together we define the specific distinct physiological response to constitutive SKN-1 activation stemming from a gain-of-function mutation in XREP-4, a regulator of WDR-23 and SKN-1, that can be leveraged to understand the age-related effects of dysregulated transcriptional homeostats.

## Discussion

NRF2, the mammalian ortholog of SKN-1, plays an orthologous role as a master regulator of antioxidant, xenobiotic, and proteostasis defense systems [6, 49–52]. Although NRF2 activation is important for maintaining homeostasis in response to both endogenous and exogenous stressors [8, 53], the activation of NRF2 can contribute to resistance to chemo- and radiation-based therapies as NRF2 acts to detoxify the cell of the drugs designed as cancer therapies [26, 54–57]. In invertebrates, gain-of-function SKN-1 (*skn-1gf*) mutants transcriptionally activate genes associated with adaptation to oxidative stress, aging and survival, and metabolism [1, 9, 14, 19, 20]. Interestingly, the chronic activation of innate immunity is pleiotropic to the animal and drives the dysregulation of cellular lipid homeostasis that results in diminished health and shortened lifespan [16, 19]. Taken together, unchecked SKN-1/NRF2 transcription can yield pleiotropic outcomes.

Several genetic screens have identified loss-of-function mutations that activate cellular phase II detoxification systems in *C. elegans*, including loss-of-function alleles of *wdr-23* [6, 21], *alh-6* [20, 29, 30, 44], but also gain-of-function alleles in *skn-1* [9, 16]. Here, we utilized whole-genome sequencing, insertion-deletion polymorphism mapping [28], RNAi phenocopy screening, and transgenic arrays to identify a novel gain-of-function mutation in the F-box protein-encoding gene *F46F11.6* (*xrep-4*) resulting in a cysteine to tyrosine amino acid change at the 283 position. F-box proteins are a family of ~326 proteins found in *C. elegans* that mediate protein-protein interactions for polyubiquitination via a ~50 amino acid motif [22, 25]. Specifically, XREP-4 is the first F-box protein to regulate stress responses via interaction with SKR-1/2, a known partner of F-box proteins that act as a regulator of ubiquitination and protein degradation [25]. Epistatic analyses previously identified WDR-23 as a downstream target of XREP-4 [22]. Together, XREP-4 interacts with SKR-1/2, upstream of SKN-1, to alter the protein stability of the negative regulator of both WDR-23 and SKN-1 [22, 25]. Our work supports this model when enhanced degradation of WDR-23 by activated XREP-4gf protein leads to the diminished turnover of SKN-1 and subsequently, the constitutive induction of SKN-1 targets like *gst-4*; thus, a new model of chronic activation of the WT SKN-1 protein.

Strains containing loss-of-function alleles in *wdr-23* (*tm1817*) and gain-of-function alleles in *skn-1* have constitutive activation of *gst-4p::GFP*, and this activation can be suppressed by RNAi targeting the *skn-1* gene [42]. Similarly, we observed the constitutive activation of *gst-4p::GFP* caused by *xrep-4gf* is suppressed by *skn-1* RNAi. Previous loss-of-function (*lf*) alleles of *xrep-4* have been characterized that reduced *gst-4p::GFP* fluorescence in the presence of acrylamide or juglone, which are known strong inducers of SKN-1 activity [25, 42, 58]. These *xrep-4lf* mutants significantly reduced expression of canonical SKN-1-dependent detoxification genes, such as *gst-4*, *gst-10*, and *gst-12*, and subsequently showed marked reduction of survival to oxidative stress [25]. Additionally, animals with overexpression (OE) of *xrep-4* displayed significant expression of detoxification genes compared to wild type animals [25]. Here we demonstrate that animals with constitutive activation of SKN-1 via either *skn-1gf* or *xrep-4gf* had an overlap of 1796 genes with increased expression associated with glutathione transferase activity, immune system process, and response to xenobiotic stimulus. Moreover, these animals displayed significant survival in response to oxidative stress.

In addition to roles in cytoprotection, SKN-1 is a regulator of lifespan and aging [1, 13, 24, 59] and XREP-4 potentially regulates additional targets beyond WDR-23 that are important for maintaining cellular and organismal homeostasis. Although *skn-1gf* and *xrep-4gf* mutants display some phenotypic differences from each other, the similar gene expression changes observed between constitutively active mutants suggest that the observed physiological responses are complex and are unlikely to be due to one specific gene, but rather a collection of genes working in concert. Moreover, diet [60, 61], particularly the HT115/K-12 bacterial diet, is a potent suppressor of *skn-1gf* metabolic outcomes[16, 19] and notably, the HT115/K-12 bacterial diet also suppresses the *xrep-4gf* age-dependent somatic depletion of fat phenotype (Figure S2A-F). Similarly, the HT115/K-12 diet partially suppresses the age-dependent changes in movement observed in both *skn-1gf* and *xrep-4gf* animals (Figure S2G-J). Overall, our work reveals that while *xrep-4gf* animals showed similar premature age-related decline in lipid homeostasis and motility, as compared to *skn-1gf* mutant animals, the phenotypes of *xrep-4gf* animals are less severe.

Taken together, our results reveal an important new regulatory mechanism for constitutive SKN-1 activation with fewer detrimental and pleiotropic consequences over the lifespan. This novel gain-of-function mutation in *xrep-4*, in conjunction with previously described models of SKN-1 regulation, define the multilayered regulation of SKN-1 activation that is important for the maintenance of redox balance, lipid homeostasis, and physiology. This new data is important as previous work has shown that ectopic overexpression of SKN-1 can increase survival, while activation of endogenous SKN-1 by *skn-1gf* mutation [9, 16, 19] and *xrep-4gf* reported here results in decreased survival. As such, this research provides an alternative to explore upstream regulators of SKN-1/NRF2 activation for health across the lifespan.

### Supplementary Information

Below is the link to the electronic supplementary material.

Supplementary file1 (DOCX 18 KB)

Supplementary file2 (XLSX 54 KB)

Supplementary file3 (XLSX 62 KB)

Supplementary file4 (XLSX 12 KB)

Supplementary file5 (XLSX 97 KB)

Supplementary file6 (XLSX 39 KB)

Supplementary file7 (TIF 9204 KB)

Supplementary file8 (TIF 7556 KB)

## Data Availability

All data are available in the main text or the supplementary materials.
