# Supplementary material for: Comparative analysis of the molecular and physiological consequences of constitutive SKN-1 activation
Source: GeroScience. 2023 Sep 26;45(6):3359–70. doi: 10.1007/s11357-023-00937-9 (PMC10643742; doi:10.1007/s11357-023-00937-9)
Supplement: Supplementary file 1 — Supplementary file1 (DOCX 18 KB) [file 11357_2023_937_MOESM1_ESM.docx]

**TABLE S1: List of non-synonymous mutations identified on Chromosome LGI.**

| **Wormbase Gene ID** | **Public Name** | Sequence name | Chromosome location (Mb) |
| --- | --- | --- | --- |
| WBGene00022032 | Y65B4A.9 | Y65B4A.9 | -18.6 |
| *WBGene00021689 | znf-236 | Y48G8AL.10 | -16.94 |
| WBGene00022139 | tub-2 | Y71G12A.3 | -12.48 |
| *WBGene00021473 | sydn-1 | Y39G10AR.17 | -8.85 |
| WBGene00022282 | Y74C10AR.2 | Y74C10AR.2 | -8.51 |
| WBGene00004946 | sop-3 | Y71F9B.10 | -7.21 |
| WBGene00021840 | pign-1 | Y54E10BR.1 | -4.5 |
| WBGene00003060 | lpd-3 | Y47G6A.23 | -3.17 |
| WBGene00016321 | tppp-1 | C32E8.3 | -2.59 |
| WBGene00017908 | F28H1.1 | F28H1.1 | -1.89 |
| WBGene00018509 | xrep-4 | F46F11.6 | 0.27 |
| WBGene00003472 | mtk-1 | B0414.7 | 0.45 |
| *WBGene00016946 | C55B7.10 | C55B7.10 | 1.17 |
| *WBGene00003041 | lin-61 | R06C7.7 | 1.86 |
| WBGene00004324 | rde-2 | F21C3.4 | 1.87 |
| WBGene00004378 | rme-8 | F18C12.2 | 2.49 |
| WBGene00013960 | cutl-8 | ZK265.8 | 2.7 |
| WBGene00009941 | F52F12.8 | F52F12.8 | 4.06 |
| WBGene00219319 | H25P06.5 | H25P06.5 | 7.94 |
| WBGene00005528 | sri-16 | F15H9.4 | 13.07 |
| WBGene00011030 | nsph-1.2 | R05D7.5 | 13.07 (interpolated) |
| WBGene00003671 | nhr-81 | C47F8.8 | 13.12 |
| WBGene00011653 | oac-43 | T09E11.4 | 13.16 |
| WBGene00004196 | prx-11 | C47B2.8 | 16.8 |
| WBGene00013509 | Y71A12B.12 | Y71A12B.12 | 22.49 |

*Mutation found in background strain
