# Supplementary figures and images for: Comparative analysis of the molecular and physiological consequences of constitutive SKN-1 activation
Source: GeroScience. 2023 Sep 26;45(6):3359–70. doi: 10.1007/s11357-023-00937-9 (PMC10643742; doi:10.1007/s11357-023-00937-9)

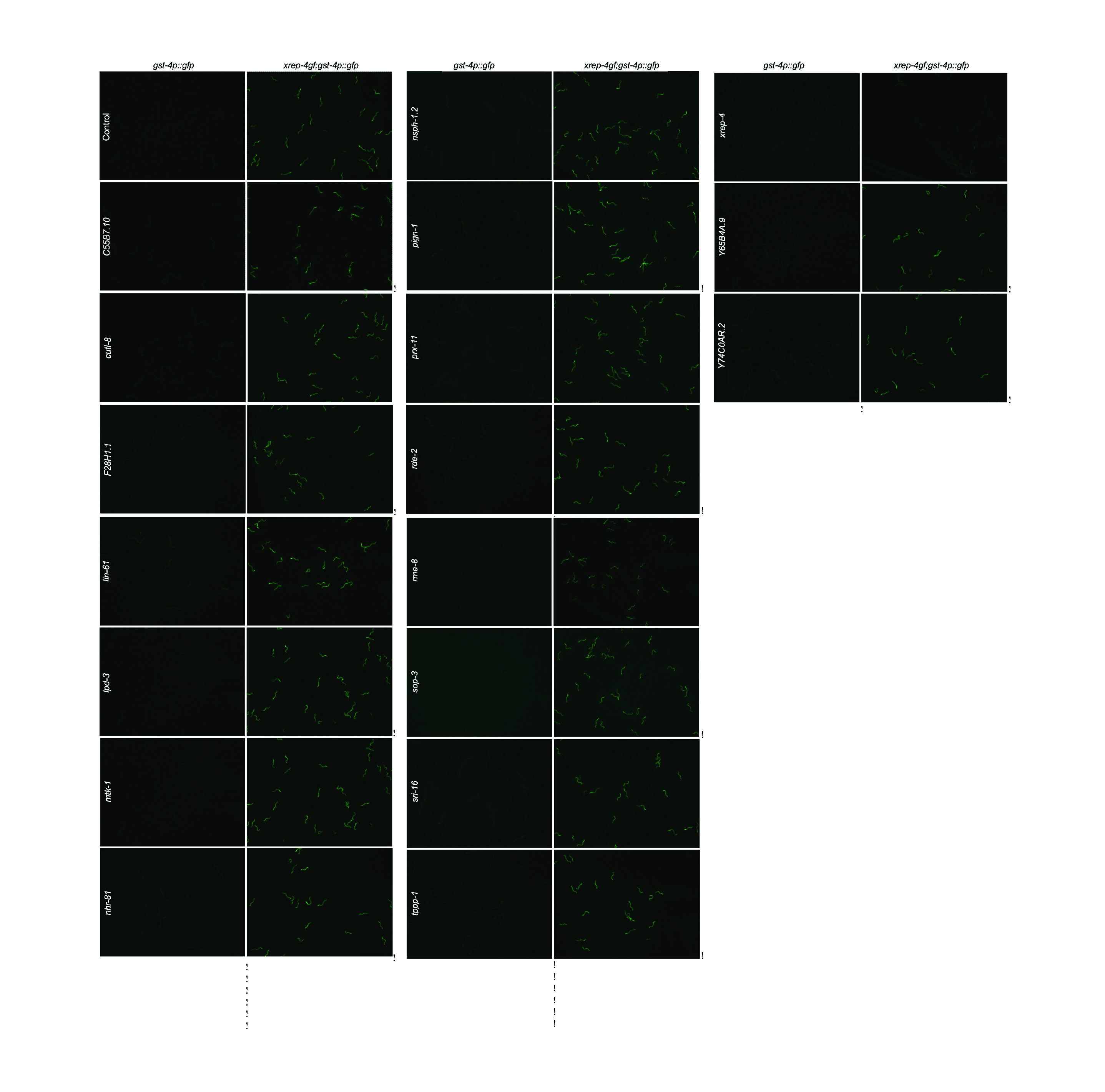

Supplement: Supplementary file 7 — Supplementary file7 (TIF 9204 KB) [file 11357_2023_937_MOESM7_ESM.tif]

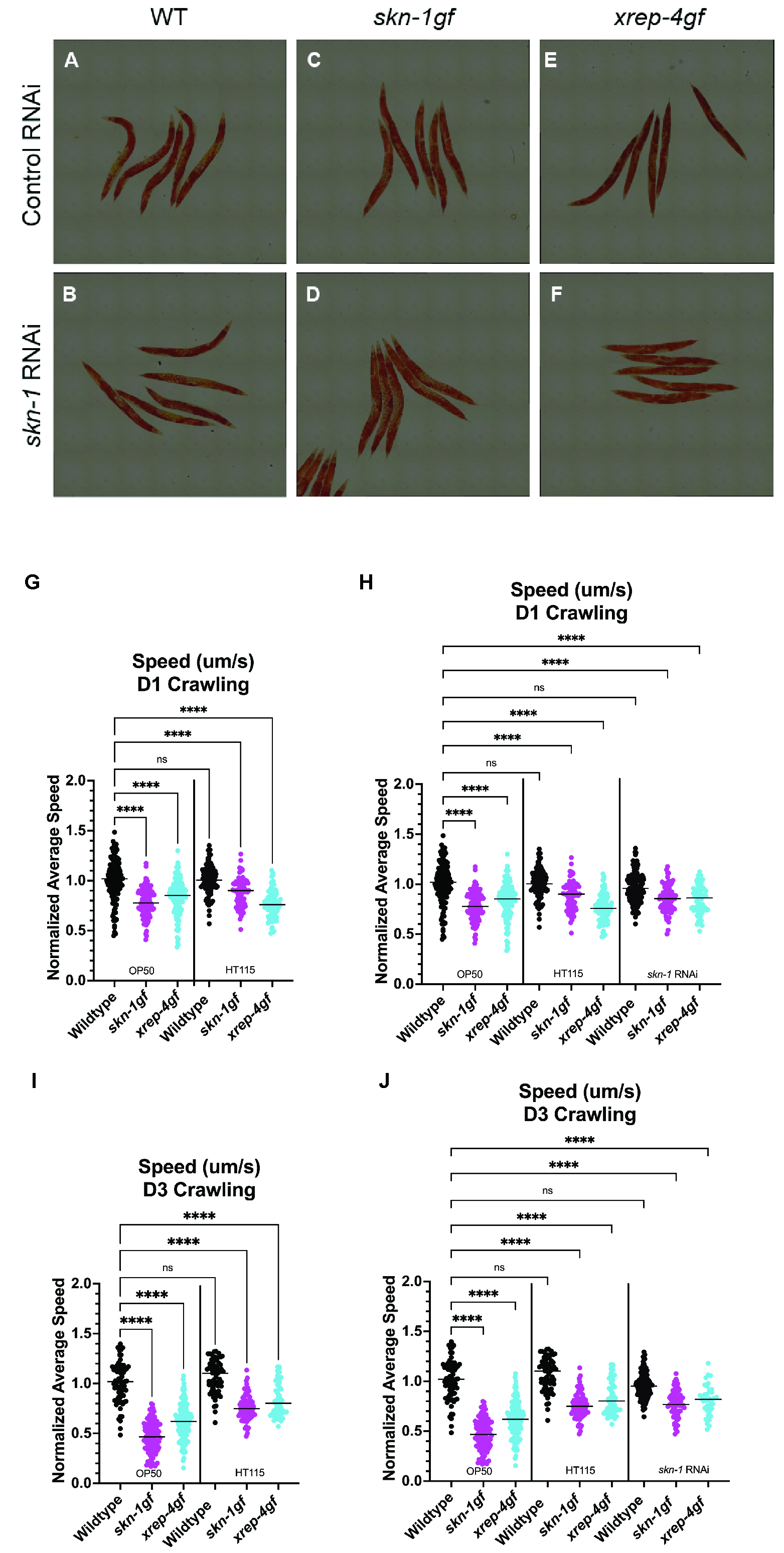

Supplement: Supplementary file 8 — Supplementary file8 (TIF 7556 KB) [file 11357_2023_937_MOESM8_ESM.tif]
